# Assessment of Occlusal Vertical Dimension Change in Mechanical and Virtual Articulation: A Pilot Study

**DOI:** 10.3390/dj10110212

**Published:** 2022-11-08

**Authors:** Yu-Chun Lin, Rebecca Scialabba, Jason D. Lee, Jie Sun, Sang J. Lee

**Affiliations:** Department of Restorative Dentistry and Biomaterials Science, Harvard School of Dental Medicine, Boston, MA 02115, USA

**Keywords:** occlusal vertical dimension, facebow transfer, virtual articulation

## Abstract

The aim of this clinical study was to investigate the change in occlusal vertical dimension (OVD) with dental casts mounted on a mechanical articulator using an average axis facebow and on a virtual articulator mounted using the Bonwill triangle and the Balkwill angle and compare these groups with OVD change observed clinically in patients. Casts were obtained from each patient (*n* = 14) and mounted on a semi-adjustable articulator in the facebow preservation group (FPG) and on a virtual articulator using average anatomic values in the average mounting group (AMG). Customized mandibular anterior splints were virtually designed at an OVD increased by 3, 6, and 9 mm. Digital buccal scans were performed with the anterior devices in the participants’ mouths in the intraoral group (IOG), AMG, and FPG at the different OVD increases accordingly. While no statistically significant differences (*p* > 0.05) were observed in the posterior interocclusal measurements with the incisal guide pin raised by 3 mm and 6 mm among all groups, a 9 mm increase resulted in a significant difference between AMG and IOG. The interocclusal posterior-to-anterior opening ratio observed clinically was 1:1.575. Increases in OVD up to 6 mm on dental casts mounted using average anatomic values performed similarly to the actual intraoral changes.

## 1. Introduction

A physiological occlusal vertical dimension (OVD) is critical for occlusal stability, functional occlusal harmony with temporomandibular joints (TMJs), patient comfort, and dentofacial esthetics. The etiology of OVD loss in dentate patients includes congenital anomalies, attrition, erosion, lack of posterior support, deep vertical overbite with insufficient horizontal overlap, and steep anterior guidance [1,2,3,4]. Restoration at an increased OVD in dentate patients is indicated to achieve adequate restorative space, reestablish ideal occlusal relationships, and harmonize dentofacial esthetics [5].

Restoration at an increased OVD is a comprehensive prosthodontic rehabilitation that requires a facebow record to transfer the spatial relationship of the maxillary arch to the transverse horizontal axis (THA) to an articulator. The THA defines an imaginary line passing through the condyles around which the mandible will rotate in the sagittal plane [6]. An average axis facebow approximates the location of the THA based on average anatomic landmarks [6]. The transfer of an average axis facebow record to a semi-adjustable articulator provides suitable accuracy for most restorations, as deviation from the true THA up to 5 mm results in negligible mandibular anteroposterior displacement [7]. However, average axis facebows may not compensate for anatomical asymmetries, which can result in mispositioned dental casts on the articulator relative to the patient’s true anatomic position and occlusal discrepancies when altering OVD [8,9].

A kinematic facebow more precisely locates the THA based on mandibular border movements [10,11]. This record is unique to each patient and is transferred to a fully adjustable articulator. This precise identification of the THA minimizes the occlusal discrepancies in altering OVD [11]. The alterations in OVD on an articulator with dental casts mounted with a kinematic facebow may most precisely represent a patient’s true arc of mandibular closure and opening [12]. However, this workflow is not commonly used for most rehabilitations, as it requires additional operator expertise and chair time.

Alternatively, another technique to transfer the relationship of the maxillary arch to the THA to an articulator circumvents the use of a facebow record by incorporating the average values of the Bonwill triangle and the Balkwill angle. W. Bonwill described a 4-inch equilateral triangle formed by lines connecting the contact point of the mandibular central incisor’s incisal edge to the midpoints of the condyles [6]. Balkwill was the first to describe the advancing condyle’s downward and forward movement and measured the average angle between the imaginary occlusal plane and the Bonwill triangle. The Bonwill triangle and the Balkwill angle became the basis for constructing contemporary dental articulators. However, the need of the average values to position maxillary casts has been controversial. Ahlers reported that the use of an arbitrary facebow significantly improved the reliability and validity of maxillary cast transfer to an articulator than a transfer using the average values in a patient simulator study [13].

With the advancement in digital dentistry, virtual articulation has emerged to relate the jaws and simulate jaw movements in three-dimensional virtual space [13,14]. One challenge of virtual articulation is the transfer of the anatomically correct position of the maxilla onto the virtual articulator [14]. Lepidi et al. classified virtual articulators into two major types: completely adjustable and mathematically simulated [15]. Completely adjustable virtual articulation reproduces exact patient-specific mandibular movements. The completely scanned arches are positioned onto a virtual articulator with patient reference based on their capabilities to transfer the patient’s maxillary arch position and functional jaw movement [14,15]. With mathematically simulated virtual articulation (MSVA), the jaw movement is mathematically simulated and arbitrarily preset based on average anatomic values, a design similar to that of a semi-adjustable, mechanical articulator. In MSVA, the arches are mounted by using the Bonwill triangle and the Balkwill angle and a digital scan of the teeth in maximum intercuspal position (MIP). MSVA is sufficient for the diagnostic phase of most cases in which the OVD will remain the same before and after treatment.

To date, there are no available data to demonstrate that altering the vertical dimension on dental casts mounted by using an average axis facebow and average anatomic values in a virtual articulator accurately reflects the true change in position of the mandible relative to the maxilla in the patient. The aim of this pilot clinical study was to investigate the accuracy of vertical dimension changes measured on casts mounted using an average axis facebow and virtually mounted using average anatomic values through a comparison with the actual intraoral measurements. The study was also to examine the ratio of posterior-to-anterior interocclusal space at increased vertical dimension. The null hypothesis was that no difference in posterior interocclusal measurement at each incisal guide pin position (3, 6, 9 mm) would be found in casts mounted by using an average axis facebow or average anatomic values of the Bonwill triangle and the Balkwill angle when compared to true vertical dimension changes observed clinically in patients.

## 2. Materials and Methods

### 2.1. Study Design

This study was approved by the Harvard Medical School Committee on Human Studies (IRB Nr. 18-0873) and followed the CONSORT 2010 statements. Fourteen participants (eight men and six women) between the ages of 25 to 35 years were enrolled. All participants presented with completely healthy dentition with dental Class I relationships, a stable occlusion, and no signs of OVD loss. Exclusion criteria included participants with gross facial asymmetry, temporomandibular joint disorders, a history of dentofacial trauma, orthognathic or plastic surgery, craniofacial or neuromuscular disorders affecting the facial form, and dental Class II and Class III relationships.

**Figure 1:**
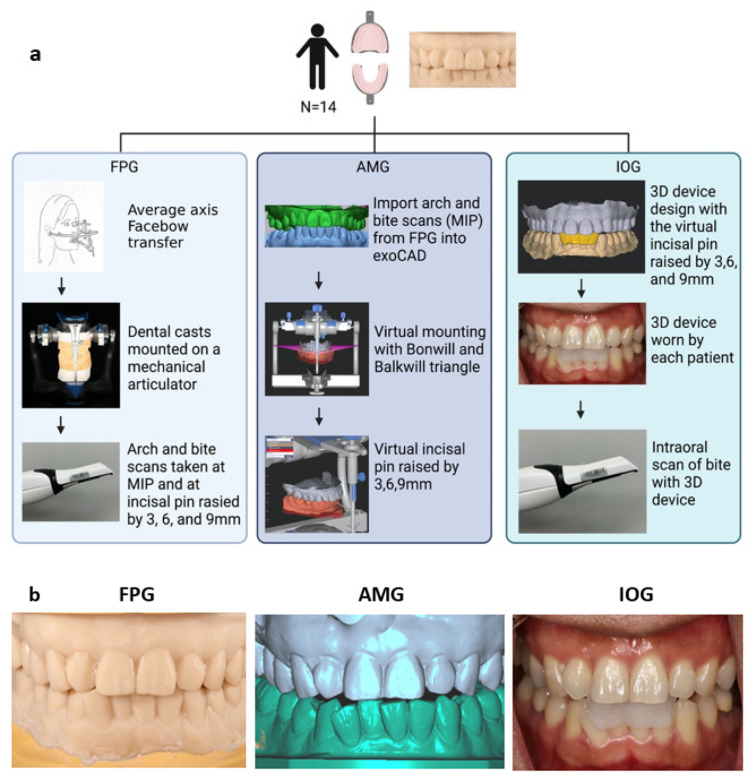

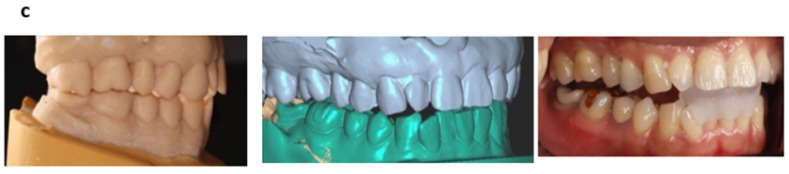
(**a**) Workflow for the facebow preservation group (FPG), average mounting group (AMG), and the intraoral group (IOG). (**b**) Anterior opening and (**c**) posterior opening when OVD + 3 mm.

Figure 1 shows the mounting techniques and the workflow employed in this study. The first group consists of the facebow preservation group (FPG), which was mounted using a mechanical articulator while the second group consisted of the average mounting group (AMG), which was mounted using a virtual articulator. A third group, the intraoral group (IOG) was formed from the AMG (Figure 1). In the FGP, impressions of each participant were made with irreversible hydrocolloid (Jeltrate; Dentsply Sirona) and centric occlusal records were obtained with polyvinyl siloxane interocclusal registration material (Exabite II NDS; GC America). The impressions were poured with a type III dental stone (Microstone; Whip Mix Corp) and the resulting casts were articulated on a semi-adjustable arcon articulator (Artex CR; Amann Girrbach AG) using an average axis facebow (Artex Rotofix; Amann Girrbach AG) record represented by the right and left external auditory meatus as the posterior reference points and a point 42 mm inferior to the nasal locator of the facebow as the third reference point formed the participants’ horizontal plane. All casts were mounted to the same articulator by a single calibrated investigator (J.S).

**Figure 2:**
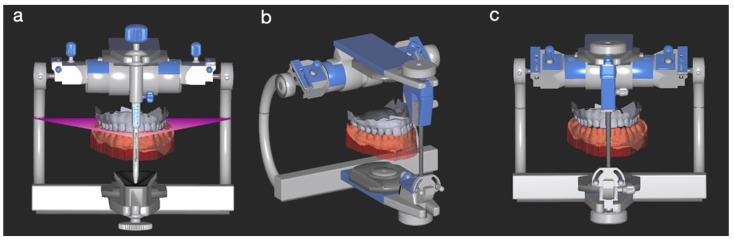
Virtual articulation of the average mounting group (AMG) in the Exocad CAD^®^ software (v3.0). (**a**) MIP, frontal view. (**b**) MIP, 45-degree view. (**c**) Virtual incisal pin raised by 3 mm.

For the AMG, an intraoral scanner (3Shape TRIOS; 3Shape A/S) was used to capture the maxillary and mandibular arches and buccal scans of the casts mounted in the FPG. The corresponding standard tessellation language (STL) files were imported into a dental digital design software (exoCAD; exocad GmbH) and mounted digitally on a virtual Artex CR type A articulator by using the average values of the Bonwill triangle and the Balkwill angle. The incisal edge of the mandibular central incisors and mesiobuccal cusp tips of the mandibular first molars were the anterior and posterior reference point for the virtual mounting, respectively (Figure 2). All virtual mountings were performed with a 10° Bennett angle, a 35° condylar angle, a 23° Balkwill angle, a 110 mm arm, and a 0 mm immediate mandibular sideshift.

For the control group, the intraoral group (IOG), three mandibular anterior devices (Figure 1) were virtually designed using the AMG in the exoCAD software program with the virtual articulator incisal guide pin raised by 3, 6, and 9 mm for each participant. Each device had a peripheral thickness of 1.5 mm covering the mandibular incisor teeth with occlusal stops opposing the palatal surfaces of the maxillary anterior teeth. The devices were 3D printed in clear resin (VisiJet M2R-CI; 3D Systems) with a 3D printer (ProJet MJP 2500 Plus; 3D Systems). The devices were tried for each participant and STL files of the buccal scans were directly obtained with a 3Shape Trios intraoral scanner.

**Figure 3:**
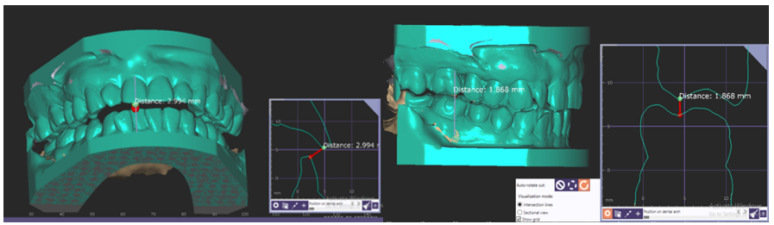
Anterior and posterior interocclusal measurements at OVD + 3 mm in the IOG.

Anterior and posterior interocclusal measurements at an increased OVD in the FPG and AMG were recorded for a comparison to the measurements obtained from the IOG. For the FPG, the incisal guide pin was raised by 3, 6, and 9 mm in the mechanical articulator and STL files of the corresponding buccal scans were obtained with the same intraoral scanner. For the AMG, the incisal guide pin was raised by 3, 6, and 9 mm in the virtual articulator and the interocclusal registrations were captured accordingly. For the IOG, the mandibular devices were tried for each participant and buccal scans were taken using the 3Shape Trios intraoral scanner. All interocclusal measurements within the FPG, AMG, and IOG were obtained and analyzed in the exoCAD software program by a single calibrated investigator (Y.L.). Anterior interocclusal measurements were taken from the midpoint of the maxillary right central incisal edge to the mandibular right central incisal edge. Posterior interocclusal measurements at each incisal guide pin position by 3, 6, 9 mm were made by determining the distance from the right maxillary first molar palatal cusp tip to its vertical projection point on the right mandibular first molar in a mid-cross-sectional view (Figure 3).

**Figure 4:**
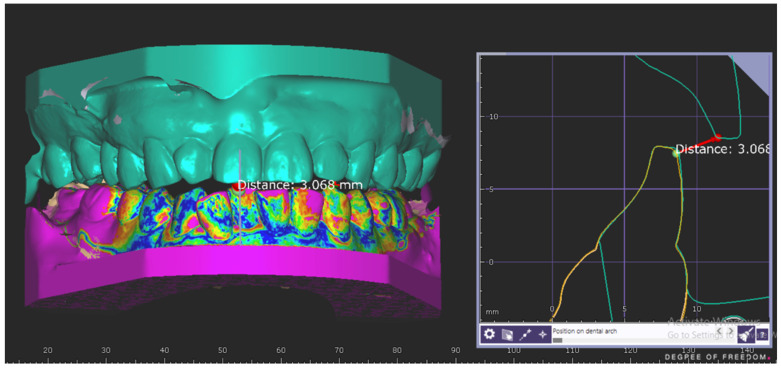
Superimposition and measurement of the anterior opening in the AMG. The mandibular arch scans of OVD + 3 mm (green) and the OVD + 6 mm (yellow) were superimposed.

To locate the precise areas of the measurements, the maxillary cast was locked in the same position throughout the measurement (Figure 4). Only the mandibular cast was superimposed using best-match alignment, and the measurements were taken comparing the different positions (3, 6, 9 mm) at the incisal pin. Triplicate values for each record were averaged. The measuring point on the maxillary incisor was the same throughout the measurements.

### 2.2. Statistical Analysis

A Kruskal–Wallis test was conducted (α = 0.05) with a statistical software program (IBM SPSS Statistics, v22; IBM Corp). A Mann–Whitney U test was used in the evaluation of any differences among the groups found during the analysis and Bonferroni correction was applied to adjust the multiple comparisons. Anterior interocclusal measurements were made by determining the distance from the midpoint of the incisal edge of the maxillary right central incisor to the midpoint of the incisal edge of the mandibular right central incisor. The ratio of posterior measurement to anterior measurement was calculated for the IOG. The correlation coefficient in linear regression was calculated to evaluate this ratio at each increase in OVD (3, 6, 9 mm).

## 3. Results

The posterior interocclusal measurements at each incisal guide pin position (3, 6, 9 mm) are presented in Table 1. From the Kruskal–Wallis test, the posterior interocclusal measurements with the incisal pin raised by 3 mm and 6 mm were not statistically significant (*p* = 0.728 and *p* = 0.101, respectively). There was a statistically significant difference with the incisal pin raised by 9 mm (*p* = 0.048). A Mann–Whitney U test showed the adjusted *p* value of 0.057 (IOG vs FPG) and 0.008 (IOG vs. AMG), respectively. Therefore, the posterior interocclusal measurements at 9 mm between the IOG and AMG were statistically different.

**Figure 5:**
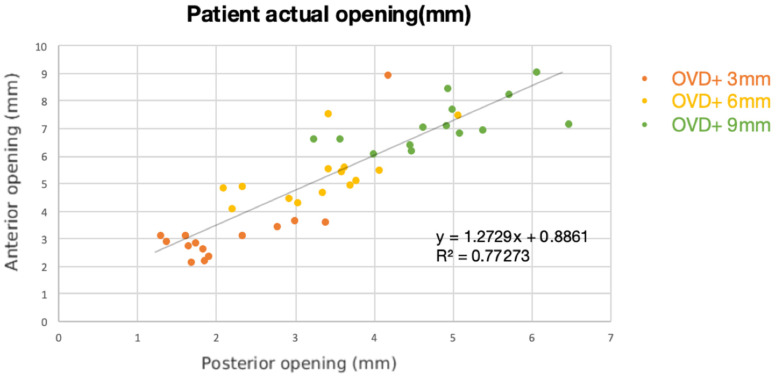
Correlation between the anterior and posterior openings in patients (IOG). X: posterior opening, Y: anterior interincisal opening. The mean posterior opening to anterior opening ratio in patients was 1:1.575.

The mean of the anterior and posterior interocclusal measurements at each incisal pin position (3, 6, and 9 mm) was recorded and demonstrated in a linear regression (Figure 5). The coefficient of determination was 0.773, which showed a high correlation of the anterior opening and posterior openings. The mean posterior opening to the anterior opening ratio clinically observed in patients was 1:1.575.

## 4. Discussion

Virtual articulation has been applied to simulate jaw movement and design dental prostheses. However, there has been limited research investigating the use of virtual articulators in complex cases involving the change in OVD and their validity and reliability to simulate jaw movement While many studies have demonstrated that OVD increase with a mechanical articulator should be limited to 5 or 6 mm, there is insufficient evidence to date to support the clinically acceptable range of the OVD change in virtual articulators [16,17].

The aim of this present pilot study was to evaluate whether the use of the Bonwill triangle and the Balkwill angle to transfer the maxillary arch position onto a virtual articulator is as valid and reliable as the transfer using an arbitrary facebow in a mechanical articulator and compare the OVD change in these groups with the OVD change observed clinically in patients.

The null hypothesis for posterior interocclusal measurements made at incisal guide pin positions of 3 mm and 6 mm was accepted as no significant differences were found between the FPG, AMG, and IOG. The null hypothesis for posterior interocclusal measurements made at the 9 mm incisal guide pin position was rejected when comparing the AMG with IOG as the measurements were statistically different at this vertical dimension.

These results demonstrate that the accuracy of the mounting techniques by facebow record and the average values can be clinically viable within an increase in OVD up to 6 mm. This finding is consistent with other studies that found that an OVD increase in both a mechanical articulator and virtual articulator should be limited to 5 or 6 mm [16,17,18]. However, minor discrepancies between the average anatomic values and the patient’s true hinge axis resulted in a greater deviation from the true position of the patient’s arches as OVD increased. It is advised that the maxillomandibular relationship is verified intraorally at the estimated OVD to minimize the discrepancies from the mounting techniques [19].

Alteration in the OVD requires an extensive diagnostic workup. The OVD opening ratio of the molars relative to the incisors has previously been reported [20,21]. Kaiser and Schelb first calculated the theoretical opening ratio of second molars, incisors, and the incisal guide pin to be 1:2:3 using a mathematical model based on simple trigonometry [20]. In this study, the opening ratio of first molars to incisors observed clinically in patients with dental Class I relationships and no OVD loss was 1:1.575. Sharon et al. [21] conducted a study to assess the opening ratio of the first molar in relation to the opening of the central incisor. They measured the average ratio to be 0.73:1 or 1:1.37. However, it is important to note that their study utilized two-dimensional digital photographs while this study utilized three-dimensional scans to calculate this ratio.

The results of this study show that average value mounting on virtual articulators can accurately represent OVD increases up to 6 mm. However, the mounting techniques of arbitrary facebow transfer to mechanical articulators and the average value mounting on virtual articulators lack sufficient accuracy and precision to simulate clinical situations in real patients when the OVD increase exceeds 6 mm, which necessitates the incorporation of alternative methods such as the use of virtual facebows or jaw tracking devices. Accurate prediction of the outcomes of OVD change is significant in the diagnostic and treatment planning phase of prosthodontic rehabilitation in determining whether occlusal adjustment or full-arch complete coverage restorations is indicated.

This clinical study has several limitations. First, the sample size was small. Second, all participants presented with a Class I dental relationship. The effects of the skeletal relationship, intercondylar distance, asymmetry of the left and right condyle, TMD, and jaw flexure on the validity of virtual articulators, and posterior-to-anterior opening ratio were not tested. Class II and Class III dental patients may present with different vertical dimension changes. Third, the anterior opening measurements were a combination of both overjet and overbite. Finally, the condylar angles and trueness to the condylar axes were not accounted for by average value virtual mountings. Future studies could include a larger and more diverse patient pool and incorporate 3D or 4D mandibular tracking devices or CBCT into the workflow to assess the vertical dimension change in different skeletal and dental relationships. Recently, a bionic jaw motion system that incorporated an optoelectronic jaw movement analyzer and a robotic device that reproduced the mandibular movement independent of hinge axis were introduced, which could be promising to accurately measure the change in the vertical dimension of occlusion and applied to a part of future study [22].

## 5. Conclusions

Within the limitations of this pilot clinical study, it was concluded that

1. The mounting techniques of the average axis facebow record and the average values using the Bonwill triangle and the Balkwill angle in a virtual articulator (AMG) can be clinically viable with an increase in OVD up to 6 mm in dental Class 1 patients.
2. The interocclusal posterior-to-anterior opening ratio observed clinically was 1:1.575, measured at the mandibular first molars and the central incisors. The coefficient of determination was 0.773, which showed a high correlation of anterior opening and posterior openings.

## Figures and Tables

**Table 1:** Mean posterior openings in mm (Mean ± SD) of facebow preservation group (FPG), average mounting group (AMG), and intraoral group (IOG).

Incisal Pin Opening (mm)	FPG	AMG	IOG	*p* Values
3	1.949 ± 0.477	1.773 ± 0.381	2.180 ± 0.845	0.728
6	3.333 ± 0.483	2.913 ± 0.578	3.354 ± 0.829	0.101
9	4.263 ± 0.636	4.080 ± 0.690	4.844 ± 0.898	0.048 *

* *p* < 0.05. *p*-value calculated with the Kruskal–Wallis test. Mann–Whitney test results: IOG vs. FPG: *p* = *0*.057; IOG vs. AMG: *p* = *0*.008 *. FPG, facebow preservation group; AMG, average mounting group; IOG, intraoral group

## Data Availability

Not applicable.

## References

[1] Schopper A.F. (1959). Loss of vertical dimension: Causes and effects: Diagnosis and various recommended treatments. J. Prosthet. Dent..

[2] Turner K.A., Missirlian D.M. (1984). Restoration of the extremely worn dentition. J. Prosthet. Dent..

[3] Abduo J., Lyons K. (2012). Clinical considerations for increasing occlusal vertical dimension: A review. Aust. Dent. J..

[4] Abduo J. (2012). Safety of increasing vertical dimension of occlusion: A systematic review. Quintessence Int..

[5] Calamita M., Coachman C., Sesma N., Kois J. (2019). Occlusal vertical dimension: Treatment planning decisions and management considerations. Int. J. Esthet. Dent..

[6] (2017). The Glossary of Prosthodontic Terms: Ninth Edition. J. Prosthet. Dent..

[7] Weinberg L.A. (1961). An evaluation of the face-bow mounting. J. Prosthet. Dent..

[8] Preston J.D. (1979). A reassessment of the mandibular transverse horizontal axis theory. J. Prosthet. Dent..

[9] Craddock F.W., Symmons H.F. (1952). Evaluation of the face-bow. J. Prosthet. Dent..

[10] Wieckiewicz M., Zietek M., Nowakowska D., Wieckiewicz W. (2014). Comparison of selected kinematic facebows applied to mandibular tracing. BioMed. Res. Int..

[11] Franklin P., McLelland R., Brunton P. (2010). An investigation of the ability of computerized axiography to reproduce occlusal contacts. Eur. J. Prosthodont. Restor. Dent..

[12] Schallhorn R.G. (1957). A study of the arbitrary center and the kinematic center of rotation for face-bow mountings. J. Prosthet. Dent..

[13] Ahlers M.O., Edelhoff D., Jakstat H.A. (2019). Reproduction accuracy of articulator mounting with an arbitrary face-bow vs. average values—A controlled, randomized, blinded patient simulator study. Clin. Oral Investig..

[14] Att W., Witkowski S., Strub J.R. (2021). Digital workflow in reconstructive dentistry. Quintessence Int..

[15] Lepidi L., Galli M., Mastrangelo F., Venezia P., Joda T., Wang H.L., Li J. (2020). Virtual articulators and virtual mounting procedures: Where do we stand?. J. Prosthodont..

[16] Inoue N., Scialabba R., Lee J.D., Lee S.J. (2022). A comparison of virtually mounted dental casts from traditional facebow records, average values, and 3D facial scans. J. Prosthet. Dent..

[17] Olthoff L.W., Van Der Glas H.W., Van Der Bilt A. (2007). Influence of occlusal vertical dimension on the masticatory performance during chewing with maxillary splints. J. Oral Rehabil..

[18] Hsu M.R., Driscoll C.F., Romberg E., Masri R. (2019). Accuracy of dynamic virtual articulation: Trueness and precision. J. Prosthodont..

[19] Rebibo M., Darmouni L., Jouvin J., Orthlieb J.D. (2009). Vertical dimension of occlusion: The keys to decision. J. Stomat. Occ. Med..

[20] Kaiser D.A., Schelb E. (1985). Geometric study of incisal guide pin opening. J. Prosthet. Dent..

[21] Sharon E., Beyth N., Smidt A., Lipovetsky-Adler M., Zilberberg N. (2019). Influence of jaw opening on occlusal vertical dimension between incisors and molars. J. Prosthet. Dent..

[22] Carossa M., Cavagnetto D., Ceruti P., Mussano F., Carossa S. (2020). Individual mandibular movement registration and reproduction using an optoeletronic jaw movement analyzer and a dedicated robot: A dental technique. BMC Oral Health.

